# Effect of anti‐microbial immunizations in the progression of amyloid pathology in a mouse model of Alzheimer's disease

**DOI:** 10.1002/alz70855_103766

**Published:** 2025-12-24

**Authors:** Catalina Valdes, Avram S Bukhbinder, Yaobin Ling, Kristofer Harris, Paul E Schulz, Rodrigo Morales

**Affiliations:** ^1^ The University of Texas Health Science Center at Houston, Houston, TX, USA, Houston, TX, USA; ^2^ The University of Texas Health Science Center at Houston, Houston, TX, USA; ^3^ University of Texas Health Science Center at Houston, Houston, TX, USA; ^4^ John P. and Kathrine G. McGovern Medical School at UTHealth, Houston, TX, USA

## Abstract

**Background:**

Recently, large epidemiological studies described those antimicrobial vaccinations were protective against AD. Whether these vaccinations prevent the deposition of amyloidosis in the brain is highly relevant to identify novel protective neuroimmune mechanisms.

**Method:**

Pfizer COVID‐19 and Fluarix vaccine were selected for this study. PBS and LPS were used as controls. Vaccine and control materials were intramuscularly administered 5 times (monthly doses) starting at 90 days old in APP/PS1 mice. Same number of male and female subjects were used. Seven days after the last treatment, a contextual behavioral test was applied to assess working memory. Aβ burden and neuroinflammatory markers were measured in brain samples and serum using histological and biochemical methods.

**Result:**

Mice treated with Pfizer and Fluarix vaccines showed improved cognitive performance compared with the control group (PBS). Importantly, mice treated with the Pfizer vaccine displayed reduced Aβ burden in hippocampus, lateral areas of the cortex, and the insular cortex compared with control subjects. On the contrary, animals treated with the Fluarix vaccine did not show significant differences. Analyses to measure peripheral and brain inflammation were also conducted.

**Conclusion:**

The stimulation of the peripheral immune system by antimicrobial immunizations could impact the brain in a positive manner. Importantly, the levels of Aβ pathology were reduced in mice treated with the Pfizer COVID‐19 vaccine. Further research in this area may provide us with valuable information about amyloidogenic and non‐amyloidogenic neuroimmune mechanisms associated with AD.

